# Effects of a Mediterranean Diet Compared with the Low-Fat Diet on Patients with Knee Osteoarthritis: A Randomized Feeding Trial

**DOI:** 10.1155/2022/7275192

**Published:** 2022-01-31

**Authors:** Alireza Sadeghi, Gholamreza Zarrinjooiee, Seyedeh Neda Mousavi, Somayae Abdollahi Sabet, Nooshin Jalili

**Affiliations:** ^1^Department of Internal Medicine, Vali-e Asr Hospital, Zanjan University of Medical Science, Zanjan, Iran; ^2^Zanjan Metaboilc Diseases Research Center, Zanjan University of Medical Sciences, Zanjan, Iran; ^3^Department of Nutrition, School of Medicine, Zanjan University of Medical Sciences, Zanjan, Iran; ^4^Department of Community Medicine, School of Medicine, Social Determinants of Health Research Center, Zanjan University of Medical Sciences, Zanjan, Iran

## Abstract

**Objectives:**

Knee osteoarthritis is a common global problem, especially in overweight and obese people. It is not clear that weight loss is a factor for pain reduction in these patients or dietary components are important. Herein, we compared the effects of Mediterranean (MD) and low-fat diet on pain, stiffness, and physical function in patients with knee osteoarthritis.

**Subjects:**

/

**Methods:**

In this randomized feeding trial, 129 patients with knee osteoarthritis were enrolled. Participants were randomly allocated to the MD (*n* = 43), low-fat diet (*n* = 43), and control group (regular diet) (*n* = 43) for 12 weeks by the blocked randomization method. Total Western Ontario and McMaster Universities Arthritis Index (WOMAC) score and its subscales and anthropometric indices were compared among the groups at the baseline and end of the study.

**Results:**

Weight and waist circumference reduction was significantly higher in the MD and low-fat diet groups compared with the regular group (*p* < 0.001 and *p* < 0.001, respectively), but there was no significant difference between the MD and low-fat diet groups (*p* = 0.2). Pain was significantly decreased in the Mediterranean-style compared with the low-fat (*p* = 0.04) and regular (*p* = 0.002) diet groups. Physical function was significantly improved in the MD compared with the regular diet group (*p* = 0.01), but had no significant difference with the low-fat one. Stiffness had no significant difference among the dietary groups.

**Conclusions:**

Pain severity was reduced in the MD group, but no significant change was reported in patients on low-fat and regular diets. It seems that dietary components in the MD, regardless of weight loss effect, are effective on pain reduction in patients with KOA. The present study was registered in the IRCT under the code of IRCT20200929048876N1.

## 1. Introduction

Osteoarthritis (OA) is the most predominant form of arthritis, affecting about 230 million people in the world [1]. Knee OA (KOA) is the main cause of disability in aged men and women [2]. There is no treatment for KOA and therapies focused on pain severity reduction with opioids, acetaminophen, and nonsteroidal anti-inflammatory drugs (NSAIDs), which have side effects [3–5]. Side effects and patient's discomforts from drug intake make us to find new therapies for pain relief.

In fact, the risk of OA is higher in overweight and obese people, and the prevalence is increasing because of aging in the communities [6]. Obesity plays an important role in the disease severity through several mechanisms. In addition to the mechanical pressure on joints, inflammatory markers secreted from white adipose tissue cause inflammatory status, which increase disease progression and pain severity [7]. On the other hand, it is reported that serum anti-inflammatory markers are reduced in OA patients [8]. The link between obesity and OA is complex and multifactorial. Chronic low-grade inflammation has been demonstrated as a key driver of the pathogenesis of OA [9]. Diet has important environmental factor effects on inflammatory status in the body [10]. High-fat diets including higher amounts of Trans and saturated fats increase risk factors and pain in OA patients [11]. High-fat diets decrease osteogenic gene expression, regardless of the oil type [12]. The Mediterranean diet (MD) emphasizes on fruits and vegetables, whole grains, healthy fats such as olive oil and nuts, fatty fish, and low-fat dairies [13]. Studies, although in small numbers, reported that OA prevalence is lower in people with high adherence to MD [14–16]. But, studies on this dietary pattern are scarce and have inconclusive results [17–19]. It is not clear that the pain reduction in KOA patients is resulted from the weight loss or other factors such as the diet's ingredients play a soothing role.

According to the research, there is no study comparing the effects of MD and low-fat diet in a weight loss program in patients with KOA.

## 2. Materials/Subjects and Methods

### 2.1. Design and Ethics

The present study is a randomized single-blinded feeding trial that was ethically approved by the ethics committee of Zanjan University of Medical Sciences, Zanjan, Iran (IR ZUMS REC 1398.426) on 18 Jan 2020. The aim of study was prescribed to each participant. The signed informed consent form was gathered before the study enrollment. All procedures were performed according to the CONSORT clinical trial guidelines for human studies. Registration number of clinical trial: the present study was registered in the IRCT under the code of IRCT20200929048876N1.

### 2.2. Patients

One hundred and twenty-nine patients with KOA between ages 40–75 yrs and 25 BMI<35 kg/m^2^ were included in the present study from February to September 2020. The disorder of participants was diagnosed less than one year ago according to the American College of Rheumatology criteria (20). Patients with the history of joint replacement, chronic diseases including inflammatory rheumatoid arthritis, cardiac, hepatic, kidney failure, cancer, acute myocardial infarct, type 1 and 2 diabetes mellitus, stroke, mental and nervous diseases, and serious injuries from injection or oral administration of corticosteroids for the past 6 month, intake of alcohol, and cigarette were excluded. Patients who are on a certain diet, with recent weight loss more than 4 kg during the last 2 month, allergic and sensitive to fish supplements, undergoing physiotherapy or had physiotherapy to reduce pain over the past month, and opioid consumption or nonsteroid anti-inflammatory drugs on a daily and continuous basis were not included in the present study. Unwillingness to follow a prescribed diet was another exclusion criterion.

### 2.3. Randomization and Interventions

Blocked randomization was used as three groups with 15-number blocks including five participants in each group. Randomization unit was person, and the random allocation software was used for this purpose. Random coded boxes were used for concealment. At this method, cans with similar weight, shape, and color, which were numbered according to the random sequences, were used. Three-day food diary (two regular days and one holiday) was gathered from each patient at the baseline. Then, patients were randomized to the three dietary groups based on 28 kcal/kg/day: (1) MD contains 35%, 50%, and 15% of calorie from fats, carbohydrates, and protein, respectively. Patients receive 27–37 gr fiber in this dietary pattern. Patients were emphasized to receive at least half of their grains from the whole grains. Patients can consume 150 gr of red meat each month. Olive oil can be used for salad and canola oil for frying. At least one serving of legumes and nuts must be included in the daily diet. Low-fat dairies can be consumed, and six glasses of water must be drunk each day. Mercury-free fish oil supplements were delivered to each patient to receive two times per week; (2) low-fat diet contains 20%, 65%, and 15% of total daily calorie from fats, carbohydrates, and protein, respectively. In this dietary pattern, patients did not receive any advice and dietary servings were determined for each patient. Patients in the control group were asked not to change their dietary pattern. Dietary interventions were pursued for 12 weeks. All the participants were asked not to change their activity and had mild walking on a flat surface for 30 min. Physical activity was assessed by the international physical activity questionnaire (IPAQ) short form. To control the patient's adherence, the nutritionist called each person at the end of each week and reminded the tips of diets. In addition, three-day food diary questionnaires were gathered at the end of each month from the patients for compliance and adherence check. Patients with compliance lower than 80% were excluded from the study.

### 2.4. Outcomes

The Western Ontario and McMaster Universities Arthritis Index (WOMAC) questionnaire was filled by an internal medicine resident to assess the patient's opinions on their pain and related problems considering their KOA. This questionnaire consists of five items for pain, two items for stiffness, and seventeenth items for physical function [20]. Visual analog scale score of pain (VAS) was recorded according to the questionnaire protocol.

### 2.5. Statistical Analysis

Statistical analyses were performed using IBM SPSS Statistics, version 25 (IBM Corp, Chicago IL, USA). All data were first tested for normality by the Kolmogorov–Smirnov test. To assess difference in sex among the groups, as a qualitative variable, the Chi-square test was used. Other quantitative variables including weight, age, WC, WOMAC components, and VAS were analyzed by the ANOVA test, followed by the post hoc analysis. Before and after differences in the assessed variables were analyzed by the paired-sample *t*-test. To adjust the effect of baseline weight of patients on the outcomes, the linear regression model was performed. Significance was considered at *p* < 0.05 in all comparisons.

## 3. Results

**Figure 1:**
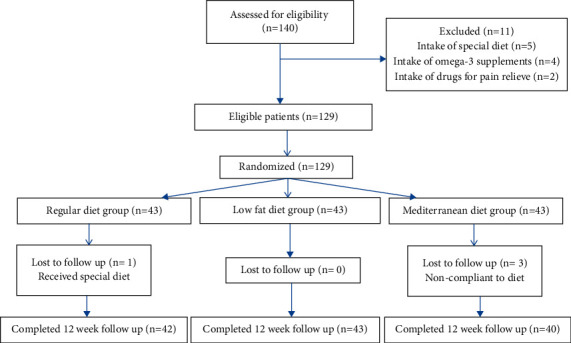
Consort flow diagram of the study from baseline up to the end.

The study protocol is illustrated in Figure 1 (Figure 1). Dietary intake before and after intervention is defined in Table 1 (Table 1).

There was no significant difference among the dietary groups on age (*p* = 0.36), sex (*p* = 0.4), pain (*p* = 0.22), stiffness (*p* = 0.84), physical function (*p* = 0.49), and visual scale analogue (*p* = 0.4) before the intervention, but weight had a significant difference among the groups at the baseline, and this difference was between the low-fat and regular diet groups (*p* = 0.04). Weight and WC were significantly decreased in the low-fat and MD groups after 12 weeks (*p* < 0.001). A significant difference was shown among the dietary groups in pain (*p* = 0.03). Pain was significantly decreased in the MD compared with the low-fat diet at the end of the study (*p* = 0.02). Pain, stiffness, physical function, and VAS were significantly reduced in all groups after intervention compared to the baseline (Table 2).

Weight and WC reduction was significantly higher in the MD and low-fat diet groups compared with the regular group (*p* < 0.001 and *p* < 0.001, respectively), but there was no significant difference between the MD and low-fat diet groups (*p* = 0.4). Pain was significantly decreased in the MD diet group compared with the low-fat (*p* = 0.04) and regular (*P* = 0.002) diet groups. Physical function was significantly improved in the MD compared with the regular diet group (*p* = 0.01), but had no significant difference with the low-fat one. Stiffness had no significant difference among the dietary groups. Pain severity (VAS) was significantly reduced in the MD compared with the regular diet group (*p* = 0.004) (Table 3).

The interaction effect between the time and diet was assessed on the WOMAC components. There was a significant effect between the low-fat diet and time on knee pain in walking (95%CI 1.1–2.29, *p* = 0.006). The knee pain in walking decreased 75% in the low-fat diet after 12 weeks of intervention, but increased 62% in the regular diet group. The knee pain in standing situation improved 100% in the MD group (95%CI 1.09–2.58, *p* = 0.01). Right and left knee pain had no significant difference among the groups, but the left knee pain was significantly decreased after dietary interventions compared with the baseline (95% CI 1.11–2.32, *p* = 0.01). Morning stiffness decreased 81% in the MD group after 12 weeks of intervention (95% CI 1.25–3.38, *p* = 0.004).

## 4. Discussion

Herein, we showed that weight and WC significantly decreased in the MD and low-fat diet groups compared with the regular diet. The pain and physical function alteration were significantly higher in the MD group compared with the regular one, but there was no significant difference between the low-fat and regular diet in these parameters. Moreover, pain reduction was significantly higher in the MD group than in the low-fat diet group. Total stiffness had no significant difference among the groups, but morning stiffness decreased 81% in the MD group at the end. Knee pain in the standing situations improved 100% in the MD group compared with others. Knee pain in walking decreased 75% in the low-fat diet group. No patient received pain-relief drugs during the study.

KOA, as a widespread complication with many physical, cognitive, and emotional side effects, is increasing due to the aging of the communities. Polypharmacy is a main problem in the aged people because of drug interactions or abuse. Therefore, there is a need to find alternate pain management approaches [21]. In a recent study, two dietary interventions including low-carbohydrate and low-fat diets were compared with a usual dietary intake over 12 weeks. Results showed that the low-carbohydrate diet reduced pain intensity and unpleasantness in some functional pain tasks, and self-reported pain was compared with the low-fat and usual diets. The low-carbohydrate diet significantly reduced oxidative stress and the adipokines compared with the other dietary groups. In the mentioned study, oxidative stress has been suggested as a cause of pain in the patients with KOA [22]. They proposed that high-carbohydrate diet produces more reactive oxygen species (ROS) in the body, which leads to more pain. Our results disagreed with the mentioned study. In the present study, pain and physical function were significantly decreased in the MD group compared with the low-fat and regular diets. We compared MD with the low-fat and regular diet on pain. MD is a diet which emphasizes on fruits and vegetables, healthy fats such as olive oil and nuts, whole grains, fish, and low-fat dairies. Sweets and red meat were reduced to one serving per month. Fiber, beta-carotene, magnesium, calcium, potassium, and omega-3 fatty acids are higher in this diet, and saturated fatty acids as well as sodium are lower. In the present study, dietary sodium and saturated fatty acids were significantly decreased; however, monounsaturated fatty acids increased in the MD group after the intervention. Pain reduction may be due to these alterations. It is proposed that this dietary pattern has anti-inflammatory effects in the body and can be as effective as NSAIDs for pain reduction [23]. Therefore, there is a need to design more high-quality experiments and trials in order to overcome pain in patients with KOA. Recent studies focused on the complementary therapies and integrative medicine for pain relief in patients with KOA. One study showed that backward walking is a pain-relief way [24]. In another study, the effect of herbal medicine was compared with NSAID therapy, as the topical application [25]. Previous studies reported the role of inflammatory markers in pain severity in KOA patients [26–28]. Decrease in salt intake in the MD decreases IFN-*γ* and regulates innate and adaptive immune mechanisms to decrease inflammation [28]. Oleocanthal is a polyphenol constituent of olive oil with strong anti-inflammatory activities [29, 30]. Previously, the extra virgin olive oil showed deleterious effects on bone in high amounts (48% of total calorie) [12]. In this context, the role of olive oil on bone and inflammatory responses need further investigations [30]. Interestingly, a randomized, double-blinded, multicentre study compared the effects of low *vs.* high doses of fish oil on the WOMAC pain score of patients with KOA at 3, 6, 12, and 24 months. Results showed that the low-dose fish oil group had greater improvement in WOMAC pain and function scores at 2 years compared with the high-dose group [31]. Decrease in inflammatory markers is related to decrease in pain, which is resulted in our study with 12 weeks of MD intervention along with weight loss. There is a significant dose-response relationship between the percentage of weight loss, and symptomatic improvement in pain of patients with KOA and weight loss is recommended as a therapeutic intervention in KOA in a community-based setting [32, 33].

Studies on the effect of MD in patients with KOA compared this dietary pattern with the usual diet. There is no study to compare the effects of MD pattern with the low-fat diet in a weight loss program. In the present study, we showed that although there was no significant difference in the amounts of weight loss between the MD and the low-fat diet, the pain severity decreased significantly in patients on the MD compared with the low-fat diet. The major limitation of the present study was the method of the dietary adherence assessment. However, the nutritionist called to each person at the end of each week for reminding the dietary tips and gathered the three-day food records; exact adherence not be measured. Moreover, outcomes were analyzed by the questionnaires that are dependent to the patient's opinion. Future studies in other dietary cultures, by considering accurate outcomes such as a radiographic test or biochemical markers, are needed to trust the results.

## 5. Conclusions

Calorie restriction, in any dietary pattern, leads to weight loss. However, weight loss had no significant difference between the low-fat and Mediterranean dietary pattern, and pain severity was reduced in the MD group. It seems that dietary components in the MD are effective on pain reduction in patients with KOA.

## Figures and Tables

**Table 1:** Dietary intake of energy and some nutrients in the three study groups at the baseline and end.

Dietary intake	Regular (*n* = 42)	Low fat (*n* = 43)	MD (*n* = 40)	*P* value^*∗*^
Total energy (Kcal)				
Before intervention	2588.2 ± 236.1	2572.9 ± 241.2	2601.5 ± 256.7	0.7
After intervention	2498.9 ± 243.9	2145.6 ± 291.2	2236.5 ± 265.8	0.4
Total carbohydrate (g/day)				
Before intervention	388.24 ± 22.3	385.9 ± 32.1	390.2 ± 28.5	0.2
After intervention	366.5 ± 25.8	321.8 ± 38.5	335.4 ± 26.1	0.2
Total protein (g/day)				
Before intervention	64.5 ± 10	64.6 ± 10.9	65.05 ± 8.9	0.102
After intervention	65.8 ± 12.1	85.1 ± 11.1	44.7 ± 7.1	0.04
Total fiber (g/day)				
Before intervention	23.8 ± 4.8	26.1 ± 2.6	25.51 ± 2.6	0.2
After intervention	23.9 ± 5.4	27.9 ± 1.76	28.1 ± 3.6	0.09
Total fat (g/day)				
Before intervention	86.3 ± 14.7	85.6 ± 12.1	86.7 ± 15.3	0.82
After intervention	85.5 ± 20.2	57.6 ± 11.5	80 ± 11.7	0.01
SFA1 (g/day)				
Before intervention	28.9 ± 5.4	29.5 ± 5.9	29.7 ± 6.8	0.3
After intervention	28.4 ± 5.6	7.9 ± 2.1	15.8 ± 4.6	<0.001
PUFA^2^ (g/day)				
Before intervention	39.8 ± 7.5	33.4 ± 5.2	39.1 ± 5.9	0.89
After intervention	41.4 ± 11.3	32.4 ± 10.1	35.5 ± 10.1	0.06
MUFA (g/day)				
Before intervention	19.1 ± 3.8	19.7 ± 1.5	18.1 ± 2.3	0.21
After intervention	19.8 ± 2.4	18.5 ± 1.7	28.7 ± 3.1	0.03
Vitamin *D* (*µ*g/day)				
Before intervention	0.63 ± 0.5	0.59 ± 0.3	0.6 ± 0.15	0.22
After intervention	0.51 ± 0.37	0.57 ± 0.5	0.59 ± 0.2	0.7
Vitamin K (*µ*g/day)				
Before intervention	101 ± 5.6	106.5 ± 7.5	105.7 ± 5.6	0.8
After intervention	110 ± 7.9	115.7 ± 5.4	120 ± 3.4	0.1
Sodium (mg/day)				
Before intervention	3250 ± 167.1	3156 ± 220.1	3278.9 ± 154.6	0.4
After intervention	3189 ± 198.5	2905 ± 121.5	2289 ± 99.5	0.07
Calcium (mg/day)				
Before intervention	793 ± 186	827 ± 186	810 ± 146	0.2
After intervention	801 ± 133	815 ± 154	850 ± 101	0.1
Magnesium (mg/day)				
Before intervention	356 ± 11.5	344.6 ± 10.8	350.1 ± 8.8	0.8
After intervention	355.6 ± 10.1	367.8 ± 15.6	410.4 ± 10	0.09

Data are expressed as means ± SD; ^*∗*^differences among groups were evaluated by the one-way ANOVA test; 1, SFA: saturated fatty acid; 2, PUFA: polyunsaturated fatty acid; 3, MUFA: monounsaturated fatty acid.

**Table 2:** Parameters at the baseline and end of the study in three dietary groups

Diets		Regular (*n* = 42)	Low fat (*n* = 43)	Mediterranean (*n* = 40)	*p* value^2^
Variables					
Age, yr	59.1 ± 9.8	57.98 ± 10.8	55.9 ± 9.5	0.36	

Sex, *n* (%)	Male	5 (12)	3 (7)	3 (7)	0.4
	Female	37 (88)	40 (93)	37 (93)	

Weight, kg	Before	68.8 ± 9.6	74.7 ± 11.4	73.2 ± 11.6	0.04
	After	69.2 ± 9.7	71.9 ± 10.5	70.2 ± 10.5	0.46
*p* value^1^		0.07	<0.001	<0.00	

WC, cm	Before	93.4 ± 4.9	95 ± 5.1	95.2 ± 7	0.28
	After	93 ± 4.8	92.5 ± 4	92.4 ± 5.9	0.83
*p* value	0.05	<0.001	<0.001		

PA, met/min/week before	3100 ± 400.9	3125.6 ± 500.1	3089.2 ± 410.6	0.28	
After	3151.2 ± 501.8	3164.3 ± 498.6	3108 ± 500.1	0.83	
*p* value		0.2	0.9	0.6	

Pain	Before	12.5 ± 3.8	13.8 ± 4	12.5 ± 3.7	0.22
	After^a^	11.8 ± 4	12.6 ± 4.4	10.2 ± 3.9	0.03
*p* value		0.01	<0.001	<0.001	

Stiffness	Before	4.4 ± 1.8	4.6 ± 2	4.3 ± 2.1	0.84
	After	4.1 ± 1.5	4.3 ± 1.9	3.7 ± 1.9	0.42
*p* value		0.01	0.001	0.006	

Physical function	Before	47.2 ± 11.9	50.2 ± 11.9	48.3 ± 11.4	0.49
	After	44.9 ± 11.8	46.5 ± 12.7	42.1 ± 11.9	0.25
*p* value		<0.001	<0.001	<0.001	

VAS	Before	6.4 ± 1.7	6.8 ± 1.9	6.8 ± 1.8	0.4
	After	5.9 ± 1.8	6.1 ± 2	5.4 ± 1.9	0.27
*p* value		0.008	<0.001	<0.001	

^a^Significant difference between the low-fat and Mediterranean diets assessed by post hoc analysis, ^1^*p* value, differences in each group from baseline up to the end assessed by a paired-sample *t*-test, ^2^*p* value, differences among the dietary groups at the baseline and the end of the study assessed by the ANOVA test, results are reported as mean± SD, p < 0.05 is significant; WC, waist circumference; PA, physical activity; VAS, visual analogue scale; MD, Mediterranean diet.

**Table 3:** Mean difference of parameters at the baseline and the end of the study in three dietary groups.

Diets	Regular	Low fat	MD	*p* value^2^
Variables	(*n* = 42)	(*n* = 43)	(*n* = 40)	
Weight, kg^a,c^	0.28 ± 0.98	−2.8 ± 1.5	−3.01 ± 2	**<0.001**
WC, cm^a,c^	−0.39 ± 1.2	−2.6 ± 1.6	−2.8 ± 1.7	**<0.001**
Pain^a,b^	−0.66 ± 1.6	−1.2 ± 1.5	−2.3 ± 2.9	**0.002**
Stiffness	−0.33 ± 0.84	−0.25 ± 0.49	−0.54 ± 1.1	0.3
Physical function^a^	−2.2 ± 3.6	−3.6 ± 5.4	−6.2 ± 9.1	**0.02**
VAS^a^	−0.43 ± 0.99	−0.74 ± 1.5	−1.3 ± 1.5	**0.005**

^a^Significant difference between the regular and Mediterranean diets assessed by post hoc analysis, ^b^significant difference between the low-fat and Mediterranean diets assessed by post hoc analysis, ^c^significant difference between the regular and low-fat diets assessed by post hoc analysis; WC, waist circumference; VAS, visual analogue scale; MD, Mediterranean diet. Bold values are significant.

## Data Availability

If additional data are needed, they are available from the corresponding author on reasonable request.
